# Pericoronary adipose tissue for predicting long-term outcomes

**DOI:** 10.1093/ehjci/jeae197

**Published:** 2024-08-06

**Authors:** Sophie E van Rosendael, Vasileios Kamperidis, Teemu Maaniitty, Michiel A de Graaf, Antti Saraste, George E McKay-Goodall, J Wouter Jukema, Juhani Knuuti, Jeroen J Bax

**Affiliations:** Department of Cardiology, Leiden University Medical Center, Albinusdreef 2, 2333ZA Leiden, The Netherlands; First Department of Cardiology, Medical School, AHEPA Hospital, Aristotle University of Thessaloniki, , St. Kiriakidi 1, Thessaloniki GR-54636, Greece; Turku PET Centre, Turku University Hospital and University of Turku, Turku, Finland; Department of Cardiology, Leiden University Medical Center, Albinusdreef 2, 2333ZA Leiden, The Netherlands; Turku PET Centre, Turku University Hospital and University of Turku, Turku, Finland; Heart Center, Turku University Hospital and University of Turku, Turku, Finland; St. Vincent’s Hospital Sydney, University of New South Wales Medical School, Sydney, NSW, Australia; Department of Cardiology, Leiden University Medical Center, Albinusdreef 2, 2333ZA Leiden, The Netherlands; Netherlands Heart Institute, Utrecht, The Netherlands; Turku PET Centre, Turku University Hospital and University of Turku, Turku, Finland; Department of Cardiology, Leiden University Medical Center, Albinusdreef 2, 2333ZA Leiden, The Netherlands

**Keywords:** atherosclerosis, coronary artery disease, coronary computed tomography angiography, major adverse cardiac event, pericoronary adipose tissue

## Abstract

**Aims:**

Pericoronary adipose tissue (PCAT) attenuation obtained by coronary computed tomography angiography (CCTA) has been associated with coronary inflammation and outcomes. Whether PCAT attenuation is predictive of major adverse cardiac events (MACE) during long-term follow-up is unknown.

**Methods and results:**

Symptomatic patients with coronary artery disease (CAD) who underwent CCTA were included, and clinical outcomes were evaluated. PCAT was measured at all lesions for all three major coronary arteries using semi-automated software. A comparison between patients with and without MACE was made on both a per-lesion and a per-patient level. The predictive value of PCAT attenuation for MACE was assessed in Cox regression models. In 483 patients (63.3 ± 8.5 years, 54.9% men), 1561 lesions were analysed over a median follow-up duration of 9.5 years. The mean PCAT attenuation was not significantly different between patients with and without MACE. At a per-patient level, the adjusted hazard ratio (HR) and 95% confidence interval (CI) for MACE were 0.970 (95% CI: 0.933–1.008, *P* = 0.121) when the average of all lesions per patient was analysed, 0.992 (95% CI: 0.961–1.024, *P* = 0.622) when only the most obstructive lesion was evaluated, and 0.981 (95% CI: 0.946–1.016, *P* = 0.285) when only the lesion with the highest PCAT attenuation per individual was evaluated. Adjusted HRs for vessel-specific PCAT attenuation in the right coronary artery, left anterior descending artery, and left circumflex artery were 0.957 (95% CI: 0.830–1.104, *P* = 0.548), 0.989 (95% CI: 0.954–1.025, *P* = 0.550), and 0.739 (95% CI: 0.293–1.865, *P* = 0.522), respectively, in predicting long-term MACE.

**Conclusion:**

In patients referred to CCTA for clinically suspected CAD, PCAT attenuation did not predict MACE during long-term follow-up.

## Graphical Abstract

**Graphical Abstract:**
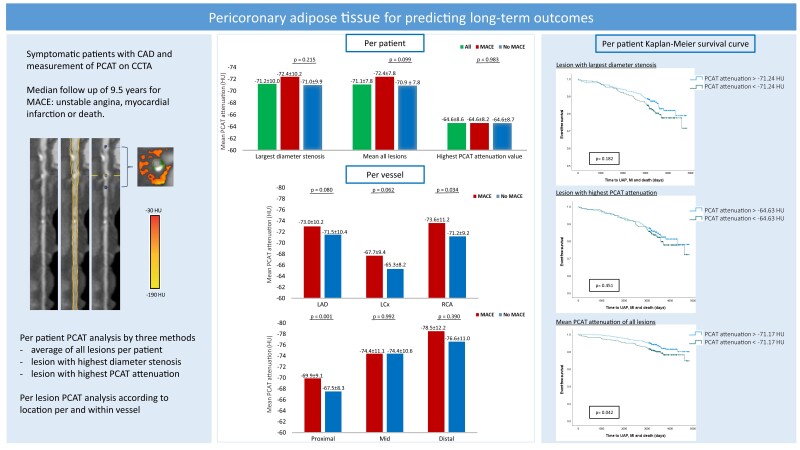

## Introduction

Non-invasive coronary computed tomography angiography (CCTA) permits the detection of atherosclerosis, including an assessment of stenosis diameter, plaque composition, and pericoronary adipose tissue (PCAT).^1^ PCAT attenuation has been shown to reflect vascular inflammation, a key element of coronary atherosclerotic plaque formation, progression, and rupture^2,3^ and is considered a non-invasive, sensitive inflammatory biomarker with the potential to improve cardiovascular risk stratification.^1,4^ Since many coronary artery plaque ruptures arise from lesions with <50% diameter stenosis,^5^ the identification of vulnerable lesions at an early stage is important. The differentiation, proliferation, and lipolysis of the adipocytes in the adjacent perivascular fat are affected by inflammation, leading to smaller adipocytes with less intracellular lipid content, which is correlated with higher PCAT attenuation values on CCTA.^1^ Significant differences in PCAT attenuation have been identified between coronary arteries with and without atherosclerosis and culprit and non-culprit lesions.^6,7^ But varying and contradictory results have been published regarding PCAT and its prognostic value for the prediction of major adverse cardiac events (MACE).^4,8–12^

Moreover, the follow-up duration in prior studies evaluating the association of PCAT with events is generally no longer than 5 years, and little is known about the degree to which PCAT attenuation is predictive of MACE at longer-term follow-up. The aim of the current study is to assess the prognostic value of PCAT attenuation for MACE in a large patient cohort with long-term follow-up.

## Methods

### Patients

A total of 922 symptomatic patients underwent CCTA at the Turku University Hospital, Turku, Finland, for suspected coronary artery disease (CAD) without previous coronary revascularization (coronary artery bypass graft or percutaneous coronary intervention) between 2007 and 2011. All patients had an intermediate pre-test likelihood of obstructive CAD. For the current analysis, 428 patients without CAD on CCTA or with only coronary artery side branch lesions were excluded. Of the remaining 494 patients, 11 had uninterpretable image quality for quantitative CCTA analysis. Overall, 1561 lesions from 483 patients with CAD were included (see Supplementary data online, *Figure S1*). This study was performed according to the Declaration of Helsinki. The study protocol was approved by the ethics committee of the Hospital District of Southwest Finland, and the need for written informed consent was waived due to an observational study design.

### CCTA image acquisition

All CCTA scans were performed with a 64-detector row positron emission tomography/CT scanner (GE Discovery VCT or GE D690, General Electric Medical Systems, Waukesha, WI, USA) as reported previously.^13^ Briefly, patients received 0–30 mg of intravenous metoprolol to achieve the target heart rate of ≤60 bpm, unless contraindicated. In addition, 800 g of sublingual nitroglycerin or 1.25 mg of isosorbide dinitrate aerosol was administered to all patients before scanning to reach maximal vasodilatation of the coronary arteries. An intravenously administered low-osmolar iodine contrast agent (48–155 mL; 320–400 mg iodine/mL) was used. To reduce radiation dose, prospectively triggered acquisition was applied if feasible.

### Quantitative plaque analysis

Anatomical evaluation was performed according to the 17-segment modified American Heart Association model, blinded to clinical and PCAT results.^14^ Coronary artery atherosclerosis was defined as any lesion ≥1 mm^2^ within or adjacent to the coronary artery lumen that could be distinguished from the surrounding pericardial tissue, epicardial fat, or vessel lumen.^15^ Each atherosclerotic plaque was graded as non-obstructive (<50% diameter stenosis), moderate (50–70% diameter stenosis), severe (70–90% diameter stenosis), or subtotal/occluded (≥90% diameter stenosis). Obstructive CAD was defined as any coronary artery plaque with ≥50% luminal diameter stenosis.

Quantitative plaque analysis was performed using dedicated software (QAngio CT Research Edition version 3.2.0.13, Medis Medical Imaging Systems, Leiden, The Netherlands).^16^ In summary, a 3D coronary tree and its side branches were extracted from the CCTA data set. Coronary arteries with a diameter of ≥1.5 mm were evaluated and automatically labelled according to vessel type and segment.^16^ Multiplanar reconstructions were created for each coronary artery with automatic detection of the lumen and vessel wall. If necessary, manual adjustments were made. Lesions in the left main artery (LM), left anterior descending artery (LAD), left circumflex artery (LCx), and right coronary artery (RCA) were included in the analysis. If the obtuse marginal artery (OM) was more dominant than the LCx, the analysis was performed in the OM. The LM was analysed as part of the LAD.

For each lesion, the software provided quantified data for location of the stenosis, stenosis severity, and plaque composition. In addition, plaque volume (PV, in mm^3^) and PV according to plaque composition were determined using predefined Hounsfield unit (HU) cut-off values: necrotic core −30 to 75 HU, fibrofatty plaque 76–130 HU, fibrous plaque 131–350 HU, and calcified plaque ≥350 HU.^17,18^ Plaque characteristics at a per-patient level were calculated by summing the PV of the different plaque components of each lesion. The mean plaque burden was calculated as PV/vessel volume × 100%.

### PCAT attenuation analysis

Evaluation of PCAT was performed with the same software (QAngio CT Research Edition version 3.2.0.13, Medis Medical Imaging Systems) in quantitatively analysed coronary lesions. PCAT was measured across the entire lesion and sampled within a radial distance from the outer vessel wall equal to the vessel diameter^1^ and calculated as the average attenuation of all voxels in the range of −190 to −30 HU.

Per-patient analyses of PCAT were performed in three ways: (i) the mean PCAT attenuation of the lesion with the most severe grade stenosis; (ii) the average PCAT attenuation of all lesions per patient; and (iii) the patient’s highest mean PCAT attenuation value (closest to −30 HU), implying the most inflamed lesion. These different methods were used, since there is no consensus on which parameter is optimal. Additionally, we tried to adjust for, as an example, multiple observations (plaques) within a single patient or vessel territory.

### Outcomes

MACE was defined as all-cause death, myocardial infarction (MI), or unstable angina pectoris, whichever occurred first. The primary outcome was the difference in the mean PCAT attenuation, measured at a per-patient level and a per-lesion level, between patients with and without MACE. Secondary outcomes were hazard ratios (HRs) for MACE. Follow-up events were identified from registry databases (Finnish National Institute for Health and Welfare and the Centre for Clinical Informatics of the Turku University Hospital) and confirmed through electronic medical records. The individual follow-up time ranged from the initial CCTA until May 2020.

### Statistical analysis

Continuous variables were presented as mean ± standard deviation (SD) when normally distributed and as median with 25–75% interquartile range when not normally distributed. Categorical data were presented as absolute numbers with percentages. For two-group comparisons of continuous variables, the independent Student’s *t*-test or Mann–Whitney *U* was used, as appropriate. For categorical variables, the *χ*^2^ test was used. In order to assess between-group differences, Bonferroni’s *post hoc* analysis was performed. Uni- and multivariable HRs with 95% confidence intervals (CIs) were calculated using Cox regression analysis to assess the association between the mean PCAT attenuation and MACE. The multivariable models were created by including age, sex, cardiovascular risk factors (family history of CAD, hypertension, diabetes mellitus, hypercholesterolaemia, and current smoking), total PV, number of lesions, and highest stenosis degree as covariates. The cumulative event-free survival rates were estimated with the Kaplan–Meier method and compared using the log-rank statistic. A two-sided *P*-value <0.05 was considered statistically significant for all tests. All analyses were performed using SPSS version 25.0 (IBM, Armonk, NY, USA).

## Results

### Patients

The study included 483 patients with CAD in at least one of the 3 major coronary arteries (54.9% male, age 63.6 ± 8.5 years) and a median follow-up duration of 9.5 years (interquartile range 8.7–10.8 years; *Table 1*). There were 59 deaths and 27 MIs at any time after enrolment, and unstable angina occurred in 14 patients. After excluding recurrent events, 88 first events were considered for analysis. Patients with MACE were older than patients without MACE (67.4 ± 8.4 vs. 62.7 ± 8.3 years, *P* < 0.001), but there were no significant differences in cardiovascular risk factors and medication use between the two groups. Patients who developed MACE more frequently had typical chest pain compared with patients without MACE (40.9 vs. 23.8%, *P* = 0.001), whereas shortness of breath was comparable between the two groups (63.5 vs. 61.8%, *P* = 0.804).

**Table 1:** Baseline demographical and clinical characteristics

	Overall cohort (*n* = 483)	Patients with MACE (*n* = 88)	Patients without MACE (*n* = 395)	*P*-value
Demographics				
Male sex, *n* (%)	265 (54.9)	55 (62.5)	210 (53.2)	0.112
Age, years	63.6 ± 8.5	67.4 ± 8.4	62.7 ± 8.3	<0.001
BMI, kg/m^2^	28.2 ± 4.8	28.4 ± 4.7	28.1 ± 4.9	0.740
Cardiac symptoms				
Typical	130 (26.9)	36 (40.9)	94 (23.8)	0.001
Atypical or non-anginal chest pain	230 (47.6)	27 (30.7)	203 (51.4)	
No chest pain	96 (19.9)	21 (23.9)	75 (19.0)	
Shortness of breath (*n* = 288)	179 (62.2)	40 (63.5)	139 (61.8)	0.804
Cardiovascular risk factors				
Diabetes mellitus	88 (21.8)	21 (29.6)	67 (20.2)	0.082
Hypertension	304 (71.9)	60 (81.1)	244 (69.9)	0.052
Hypercholesterolaemia	341 (80.4)	56 (78.9)	285 (80.7)	0.718
Family history for CAD	227 (63.9)	36 (64.3)	191 (63.9)	0.954
Current smoking	70 (16.2)	12 (16.2)	58 (16.2)	0.990
Cardiovascular medication				
Aspirin	289 (68.6)	53 (67.9)	236 (68.8)	0.883
Beta-blocker	250 (59.1)	53 (66.3)	197 (57.4)	0.149
ACE-I/ARB	184 (43.8)	40 (51.3)	144 (42.1)	0.140
Statin	244 (58.7)	40 (52.6)	204 (60.0)	0.238
Diuretic	106 (25.7)	23 (26.1)	83 (21.0)	0.386
Laboratory findings				
Total cholesterol, mmol/L	4.9 ± 1.1	4.7 ± 1.2	4.9 ± 1.0	0.131
Low-density lipoprotein, mmol/L	2.7 ± 0.9	2.6 ± 1.0	2.7 ± 0.9	0.439
High-density lipoprotein, mmol/L	1.5 ± 0.5	1.4 ± 0.4	1.5 ± 0.5	0.107
Triglycerides, mmol/L	1.6 ± 1.0	1.5 ± 0.9	1.6 ± 1.1	0.600
Creatinine, μmol/L	77.1 ± 15.2	79.7 ± 16.9	76.5 ± 14.8	0.074

Values are mean ± SD or *n* (%), as appropriate.

ACE-I, angiotensin-converting enzyme inhibitor; ARB, angiotensin receptor blocker; BMI, body mass index.

### Per-patient quantitative plaque analysis

The CCTA results of the overall cohort, stratified by the occurrence of MACE, are depicted in *Table 2*. The mean coronary artery diameter stenosis of the total cohort was 38.5 ± 20.4%. Patients who experienced MACE had more often obstructive stenosis (38 vs. 23%, *P* = 0.004) on CCTA. In addition, patients with MACE had a higher total PV and PV of any plaque compositional subtypes [total PV: 270.2 (126.2–492.1) vs. 140.1 (68.7–292.3) mm^3^, *P* < 0.001; calcified PV: 57.7 (14.8–174.4) vs. 25.1 (3.9–75.3) mm^3^, *P* < 0.001; fibrous PV: 122.2 (50.3–234.4) vs. 71.4 (36.1–128.9) mm^3^, *P* < 0.001; fibrofatty PV: 44.7 (19.4–84.0) vs. 26.2 (13.7–50.1) mm^3^, *P* < 0.001; and necrotic core PV: 29.6 (9.6–46.9) vs. 16.1 (7.1–33.7) mm^3^, *P* = 0.001].

**Table 2:** Per-patient quantitative CCTA and PCAT attenuation results

	Overall cohort *n* = 483	Patients with MACE *n* = 88	Patients without MACE *n* = 395	*P*-value
**Stenosis severity**				
Diameter stenosis, %	38.5 ± 20.4	44.8 ± 20.6	37.1 ± 20.1	0.002
Non-obstructive (0–50%), *n*	359 (74.6)	54 (62.1)	305 (77.4)	0.015
Moderate (50–70%), *n*	79 (16.4)	23 (26.4)	56 (14.2)	
Severe (70–90%), *n*	25 (5.2)	7 (8.0)	18 (4.6)	
Subtotal/occluded (≥90%), *n*	18 (3.7)	3 (3.4)	15 (3.8)	
Obstructive stenosis (≥50%), *n*	122 (25.4)	33 (37.9)	89 (22.5)	0.004
**Plaque components, mm^3^**				
Total plaque volume	158.6 (72.4–334.9)	270.2 (126.2–492.1)	140.1 (68.7–292.3)	<0.001
Calcified plaque volume	28.3 (4.88–87.99)	57.7 (14.8–174.4)	25.1 (3.9–75.3)	<0.001
Fibrous plaque volume	75.7 (37.5–154.3)	122.2 (50.3–234.4)	71.4 (36.1–128.9)	<0.001
Fibrofatty plaque volume	28.9 (14.2–55.1)	44.7 (19.4–84.0)	26.2 (13.7–50.1)	<0.001
Necrotic core plaque volume	17.7 (7.7–35.6)	29.6 (9.6–46.9)	16.1 (7.1–33.7)	0.001
**Pericoronary adipose tissue attenuation, HU**				
Largest diameter stenosis	−71.2 ± 10.0	−72.4 ± 10.2	−71.0 ± 9.9	0.215
Mean all lesions	−71.1 ± 7.8	−72.4 ± 7.8	−70.9 ± 7.8	0.099
Lesion with highest PCAT attenuation	−64.6 ± 8.6	−64.6 ± 8.2	−64.6 ± 8.7	0.983

Values are median (interquartile range), mean ± SD, or *n* (%), as appropriate.

### Per-patient PCAT analysis

Per-patient analysis of PCAT showed the mean attenuation values for the overall cohort of −71.2 ± 10.0 HU when only the lesion with the highest diameter stenosis per individual was measured (*Table 2*). An evaluation of the average of all lesions per patient showed PCAT attenuation values of −71.1 ± 7.8 HU for the total cohort, and an assessment of only the lesions with the highest PCAT attenuation demonstrated PCAT attenuation values of −64.6 ± 8.6 HU.

**Figure 1:**
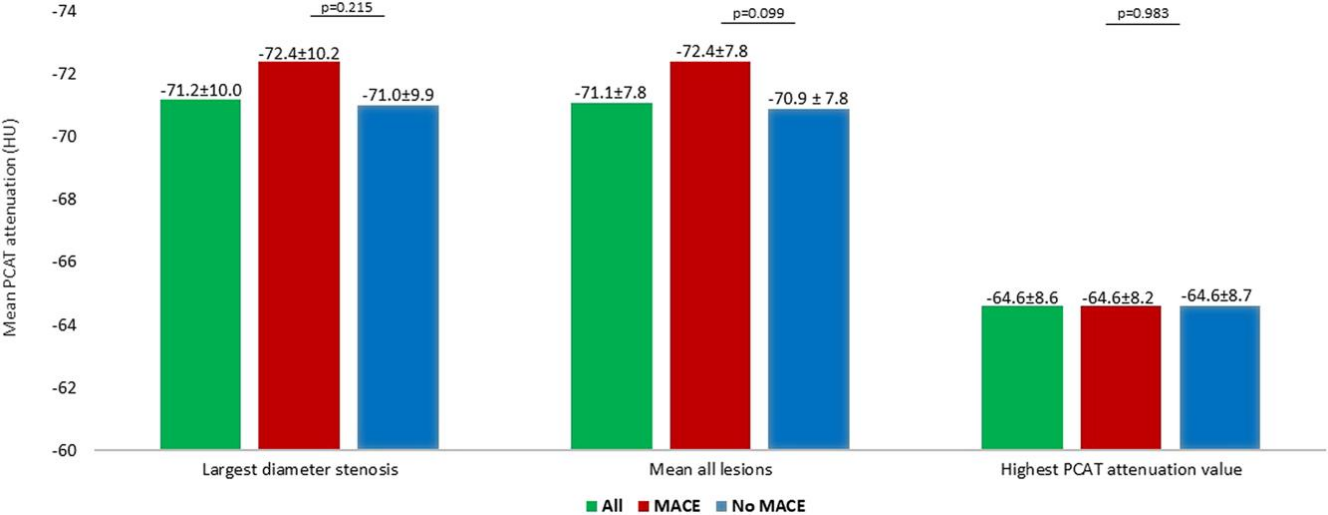
A bar chart demonstrating the mean PCAT attenuation per-patient level analyses. Values in HUs are presented as mean ± SD.

A comparison of PCAT attenuation between patients with and without MACE showed no significant differences in all the three different analysis methods (*Figure 1*). An analysis of lesions with the highest diameter stenosis showed PCAT attenuation values of −72.4 ± 10.2 vs. −71.0 ± 9.9 HU (*P* = 0.215) in patients with and without MACE, respectively. PCAT attenuation values calculated as the average of all lesions per patient were −72.4 ± 7.8 HU for patients with MACE vs. −70.9 ± 7.8 HU for patients without MACE (*P* = 0.099), and an evaluation of only lesions with the highest PCAT attenuation per patient showed very similar results for patients with and without MACE: −64.6 ± 8.2 vs. −64.6 ± 8.7 HU (*P* = 0.983).

### Per-lesion quantitative plaque analysis

The CCTA results per lesion (*n* = 1561) are stated in *Table 3*. Lesion length and plaque burden were larger in lesions of patients with MACE [9.6 (6.2–15.7) vs. 8.7 (5.3–14.3) mm, *P* = 0.006, and 48.4 ± 11.8 vs. 47.0 ± 11.2%, *P* = 0.028, respectively]. In addition, these lesions had a higher total PV and PV of calcified and fibrous plaque, compared with the lesions of patients without MACE [PV: 70.7 (36.8–127.2) vs. 56.8 (31.9–101.1) mm^3^, *P* < 0.001; fibrous PV: 32.7 (17.3–54.9) vs. 27.8 (15.4–47.1) mm^3^, *P* = 0.004; and calcified PV: 15.7 (4.9–44.7) vs. 9.2 (1.8–27.4) mm^3^, *P* < 0.001].

**Table 3:** Per-lesion quantitative CCTA and PCAT attenuation results

	All lesions *n* = 1561	Lesions in patients with MACE *n* = 355	Lesions in patients without MACE *n* = 1206	*P*-value
**General**				
Maximal diameter stenosis, %	29.5 ± 17.2	32.3 ± 17.5	28.6 ± 17.1	<0.001
Plaque volume, mm³	59.0 (33.2–108.3)	70.7 (36.8–127.2)	56.8 (31.9–101.1)	<0.001
Lesion length, mm	8.9 (5.5–14.6)	9.6 (6.2–15.7)	8.7 (5.3–14.3)	0.006
Minimal lumen diameter, mm	2.25 ± 0.88	2.2 ± 0.9	2.3 ± 0.9	0.063
Minimal lumen area, mm²	4.79 (2.89–7.44)	4.36 (2.56–7.51)	4.90 (2.98–7.40)	<0.001
Mean plaque burden, %	47.3 ± 11.0	48.4 ± 11.8	47.0 ± 11.2	0.028
**Distribution of lesions between vessels**				
RCA, *n*	425 (27.2)	108 (30.4)	317 (26.3)	0.067
LAD, *n*	897 (57.5)	185 (52.1)	712 (59)	
LCx, *n*	239 (15.3)	62 (17.5)	177 (14.7)	
**Distribution of lesions within vessel**				
Proximal, *n*	905 (58)	198 (55.8)	707 (58.6)	0.586
Mid, *n*	515 (33)	125 (35.2)	390 (32.2)	
Distal, *n*	141 (9)	32 (9)	109 (9)	
**Per-lesion plaque components, mm³**				
Calcified plaque volume	10.7 (2.2–30.3)	15.7 (4.9–44.7)	9.2 (1.8–27.4)	<0.001
Fibrous plaque volume	28.3 (15.8–48.6)	32.7 (17.3–54.9)	27.8 (15.4–47.1)	0.004
Fibrofatty plaque volume	10.1 (5.2–16.9)	11.2 (5.6–17.7)	8.8 (5.1–16.6)	0.096
Necrotic core volume	5.8 (2.7–10.6)	6.0 (3.1–11.2)	5.8 (2.6–10.4)	0.142
**Pericoronary adipose tissue attenuation, HU** **Vessel location**				
RCA (*n* = 425)	−71.8 ± 9.8	−73.6 ± 11.2	−71.2 ± 9.2	0.034
LAD (*n* = 897)	−71.8 ± 10.4	−73.0 ± 10.2	−71.5 ± 10.4	0.080
LCx (*n* = 239)	−65.9 ± 8.6	−67.7 ± 9.4	−65.3 ± 8.2	0.062
**Within-vessel location**				
Proximal (*n* = 905)	−68.0 ± 8.6	−69.9 ± 9.1	−67.5 ± 8.3	0.001
Mid (*n* = 515)	−74.4 ± 10.7	−74.4 ± 11.1	−74.4 ± 10.6	0.992
Distal (*n* = 141)	−77.0 ± 11.2	−78.5 ± 12.2	−76.6 ± 11.0	0.390

Values are median (interquartile range), mean ± SD, or *n* (%), as appropriate.

ACE-I, angiotensin-converting enzyme inhibitor; ARB, angiotensin receptor blocker; BMI, body mass index.

### Per-lesion PCAT analysis

PCAT attenuation per lesion was normally distributed for all three coronary arteries, with mean PCAT attenuation values for the RCA, LAD, and LCx of −71.8 ± 9.8, −71.8 ± 10.4, and −65.9 ± 8.6 HU, respectively (*Table 3*). Counterintuitively, higher PCAT attenuation values were seen in the lesions in the RCA of the patients without MACE compared with those who experienced MACE (−71.2 ± 9.2 vs. −73.6 ± 11.2 HU, *P* = 0.034). The analyses of the LAD and LCx showed no significant differences between groups.

**Figure 2:**
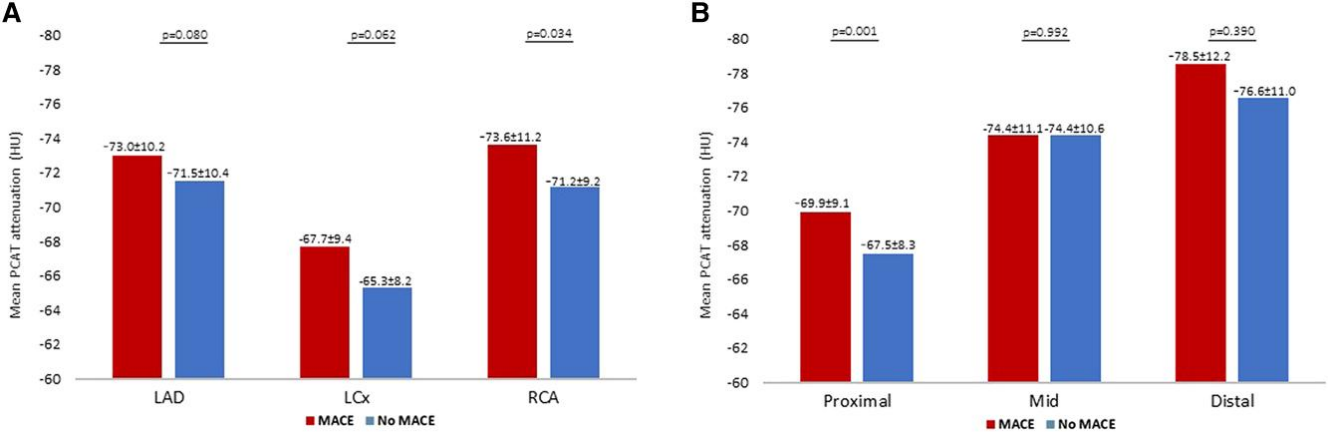
A bar chart demonstrating the mean PCAT attenuation among coronary lesions. The mean PCAT attenuation per lesion stratified by vessel (*A*) and location within vessel (*B*) of patients with MACE vs. those without MACE. Values in HUs are presented as mean ± SD.

When stratifying the lesions according to the location within the vessel, there is a significant difference in the PCAT attenuation between patients with and without MACE in the proximal lesions, with unexpectedly higher PCAT values for patients without MACE (MACE: −69.9 ± 9.1 vs. without MACE: −67.5 ± 8.3 HU, *P* = 0.001; *Figure 2*). Mid and distal lesions were not significantly different between groups.

Furthermore, subanalysis of the overall cohort showed a significant difference in PCAT attenuation based on location within the vessel, with lower PCAT attenuation values more distally in the coronary artery (proximal vs. mid *P* < 0.001, proximal vs. distal *P* < 0.001, and mid vs. distal *P* = 0.010). When dividing the cohort based on the occurrence of MACE, a significant difference in PCAT attenuation between the proximal and mid lesions and proximal and distal lesions was observed in both groups (see Supplementary data online, *Figure S2*).

### Prediction of MACE based on PCAT attenuation

Univariable Cox regression analyses per patient showed no positive association of PCAT attenuation with MACE, when calculated in three different ways: analysis of the average PCAT of all lesions per patient, the lesion with the highest obstruction in the individual, and analysis of the lesion with the highest PCAT attenuation value [HR: 0.977 (0.952–1.003), *P* = 0.084, HR: 0.987 (0.967–1.008), *P* = 0.212, HR: 1.000 (0.976–1.024), *P* = 0.973, respectively] (*Table 4*). After adjustment for age, sex, cardiovascular risk factors, total PV, number of lesions, and stenosis severity, these remained non-significant [HR: 0.970 (0.933–1.008), *P* = 0.121, HR: 0.992 (0.961–1.024), *P* = 0.622, HR: 0.981 (0.946–1.016), *P* = 0.285, respectively].

**Table 4:** PCAT as a predictor of MACE—per-patient analysis

	MACE HR (95% CI)	*P*-value
**Measure method**		
Mean PCAT of all lesions Mean PCAT of all lesions^a^	0.977 (0.952–1.003) 0.970 (0.933–1.008)	0.084 0.121
Highest obstruction lesion Highest obstruction lesion^a^	0.987 (0.967–1.008) 0.992 (0.961–1.024)	0.212 0.622
Highest PCAT values in patients Highest PCAT values in patients^a^	1.000 (0.976–1.024) 0.981 (0.946–1.016)	0.973 0.285

^a^Adjusted for age, sex, cardiovascular risk factors, PV, diameter stenosis, and number of lesions.

Per-lesion analyses showed no statistically significant HRs for PCAT attenuation with MACE for all lesions or stratified by vessel location [HR for all lesions: 0.997 (0.978–1.018), *P* = 0.797, HR for RCA: 1.024 (0.964–1.088), *P* = 0.435, HR for LAD: 0.994 (0.970–1.018), *P* = 0.602, HR for LCx: 0.975 (0.935–1.016), *P* = 0.230] (*Table 5*). The adjusted HRs with 95% CIs for all lesions and divided by vessel location were also not significant [HR all lesions: 0.974 (0.946–1.002), *P* = 0.071, HR RCA: 0.957 (0.830–1.104), *P* = 0.548, HR LAD: 0.989 (0.954–1.025), *P* = 0.550, HR LCx: 0.739 (0.293–1.865), *P* = 0.552] (*Table 5*).

**Table 5:** PCAT as a predictor of MACE—per-lesion analysis

	MACE HR (95% CI)	*P*-value
All lesions All lesions^a^	0.997 (0.978–1.018) 0.974 (0.946–1.002)	0.797 0.071
**Vessel location**		
RCA RCA^a^	1.024 (0.964, 1.088) 0.957 (0.830–1.104)	0.435 0.548
LAD LAD^a^	0.994 (0.970, 1.018) 0.989 (0.954–1.025)	0.602 0.550
LCx LCx^a^	0.975 (0.935, 1.016) 0.739 (0.293–1.865)	0.230 0.522

^a^Adjusted for age, sex, cardiovascular risk factors, PV, and diameter stenosis.

The Kaplan–Meier survival curves are shown in Supplementary data online, *Figure S3*. No difference in event-free survival was observed in lesions with the largest diameter stenosis and the highest PCAT attenuation when dichotomized according to their mean.

Analysis of the mean PCAT of all lesions per patient showed an event-free survival rate of 79.9% for patients with the mean PCAT attenuation >−71.17 HU vs. an event-free survival rate of 69.2% for patients with the mean PCAT attenuation <−71.17 HU (log-rank *P* = 0.042).

## Discussion

The current study showed that in patients with an intermediate pre-test likelihood of obstructive CAD, PCAT attenuation derived from CCTA was not associated with MACE over a median follow-up duration of 9.5 years (interquartile range 8.7–10.8 years).

Perivascular adipose tissue assessed from CCTA has recently been identified as a marker of local coronary artery inflammation, and previous studies have demonstrated a relation between PCAT, inflammation, and atherosclerosis.^1,19,20^ Inflammation is important in coronary artery plaque formation and plaque rupture.^2,3,21^ It alters the morphological and functional characteristics of the PCAT, leading to smaller adipocytes and lower lipid content, with a subsequently increased aqueous component. This can be measured non-invasively on CCTA as higher PCAT attenuation and has been considered as a parameter improving cardiovascular risk stratification.

Prior studies investigated the predictive value of PCAT for MACE and reported varying results.^4,8,11,12,22,23^ In the CRISP-CT^4^ and the ORFAN studies,^12^ a proprietary algorithm was used to incorporate PCAT attenuation in the calculation of the fat attenuation index (FAI). The findings in the CRISP-CT study demonstrated that high perivascular FAI values were related to increased cardiovascular mortality. In addition, evaluation of the FAI of the RCA added incremental value to cardiac risk prediction. The ORFAN study found that an increased FAI score in any coronary artery was associated with a higher risk of MACE, independent from risk factors and plaque burden. Van Diemen *et al*.^8^ demonstrated that PCAT attenuation around the RCA was not associated with outcomes as strongly as plaque burden-related CCTA parameters and ischaemia, but still showed additional prognostic value beyond clinical and quantitative plaque characteristics and ischaemia. Contrarily, a recent study by Wen *et al*.^10^ observed no incremental prognostic value of RCA PCAT attenuation in predicting MACE beyond the Coronary Artery Disease Reporting and Data System. In addition, no association was found in high-risk patients, referred for invasive coronary angiography with known or suspected CAD, for PCAT with MACE in any of the three coronary arteries.^11^ The included patients in these studies vary widely and the follow-up time did not exceed 5 years. The current study examined the predictive value of PCAT in an intermediate risk cohort with a follow-up duration of >5 years. PCAT attenuation was not an adequate prognostic marker for events in the current study and may be partially explained by long-term follow-up. It is possible that increases in PCAT attenuation only arise a short period before cardiovascular events and therefore potential future culprit lesions are difficult to detect on CCTA. Furthermore, the current study includes patients with an intermediate risk of obstructive CAD, which distinguishes this study from other studies including higher-risk patients in whom a significant difference between the culprit and non-culprit lesions was observed.^7,24^ The significant difference in PCAT attenuation of proximal lesions and lesions in the RCA between patients with and without MACE, with counterintuitively higher PCAT attenuation noted in patients without MACE, as well as the lower event-free survival in patients with lower mean PCAT attenuation, is of doubtful biomedical significance and may simply be due to random variation and multiple testing. There is no gold standard regarding the measurement and evaluation of PCAT attenuation, and various methods are described in previous research.^7,25–30^ Most frequently, the analysis of the proximal part of all three coronary arteries, or the RCA only, is used. Per-lesion level analysis is another option, which can include evaluation of specific lesions such as culprit and non-culprit, the lesion with the most severe stenosis, or all lesions per patient.

In addition, certain image acquisition and patient characteristics might be considered as confounding parameters when evaluating PCAT. Considering technical factors, significant differences in the mean PCAT attenuation based on the CT scanner type used have been demonstrated in prior studies, and positive associations were found for PCAT with tube voltage and current, and pixel spacing.^6,8,9^ The patients’ heart rate has been shown to influence PCAT attenuation as well, and after adjustment for these patient and imaging characteristics, Boussoussou *et al*.^9^ determined that the association between PCAT and non-calcified plaque did not hold. There is a large variability within studies regarding the adjustment for possible confounding factors. The CRISP-CT^4^ and ORFAN studies^12^ both assessed the FAI using an algorithm incorporating PCAT attenuation and artificial intelligence.^31^ As PCAT is driven by multiple factors, the developers state that the algorithm is able to adjust for anatomical, biological, and technical factors.^32^ Both studies demonstrated positive results regarding the predictive value of perivascular adipose tissue for MACE, potentially the result of the assessment of FAI, instead of solely PCAT attenuation.

Furthermore, this study showed lower PCAT attenuation for distal lesions compared with proximal lesions. This is most probably caused by the lower contrast density in the distal region compared with the proximal region, affecting the PCAT attenuation surrounding the vessel.^33^ Analyses performed on a per-lesion level should consider taking into account the location of the lesion. The differences in PCAT attenuation values are relatively small and affected by many factors. More research is needed to better understand which factors influence this parameter and how to adjust for them. In addition, the association of PCAT with atherosclerosis and cardiovascular events and the optimal way to implement this parameter clinically are poorly understood and further research is needed.

### Limitations

This is a single-centre, retrospective observational study. The observational design of the study has inherent limitations including selection bias and unmeasured confounding. In addition, details regarding image acquisition and patients’ heart rate were incomplete, and therefore, adjustment for these factors was not performed. Information on the causes of death was not available, and therefore, the metric of all-cause mortality was used.

## Conclusion

PCAT attenuation values were derived from CCTA images of patients with clinically indicated CCTA and a median follow-up duration of 9.5 years. PCAT attenuation was not positively associated with MACE on both a per-lesion and a per-patient level and did not predict outcomes. Further research is needed using different cohorts to investigate the association of PCAT with atherosclerosis and cardiovascular events.

## Supplementary data

Supplementary data are available at *European Heart Journal - Cardiovascular Imaging* online.

## Supplementary Material

jeae197_Supplementary_Data

## Data Availability

The data sets generated during and/or analysed during the current study are available from the corresponding author on reasonable request.
