# Supplementary material for: Pericoronary adipose tissue for predicting long-term outcomes
Source: Eur Heart J Cardiovasc Imaging. 2024 Aug 6;25(10):1351–9. doi: 10.1093/ehjci/jeae197 (PMC11441029; doi:10.1093/ehjci/jeae197)
Supplement: jeae197_Supplementary_Data [file jeae197_supplementary_data.docx]

**SUPPLEMENTAL Figure 1.**Flow chart of patient selection.


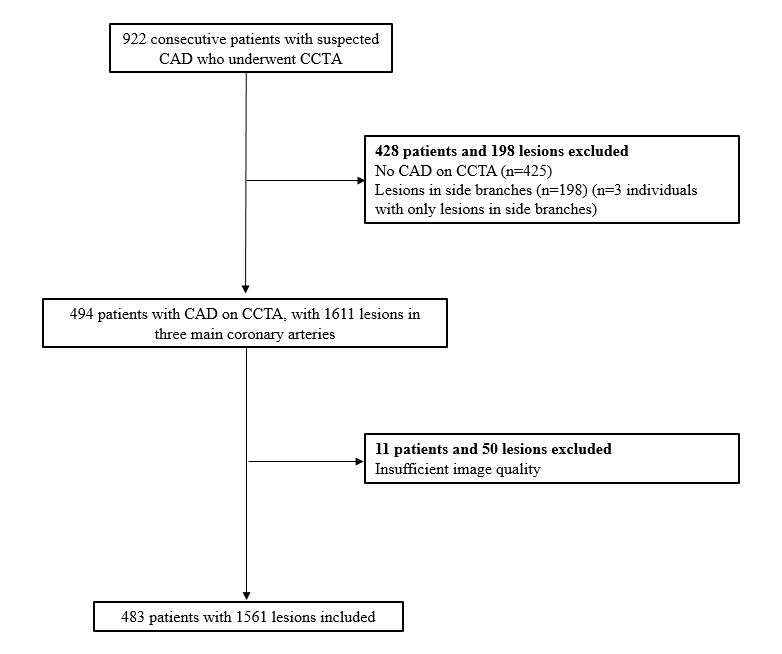


**SUPPLEMENTAL Figure 2.
Bar chart demonstrating the mean PCAT attenuation among coronary lesions stratified by location within vessel.**Mean PCAT attenuation per lesion stratified by vessel (A) and within vessel location (B) of patients who developed MACE vs. without MACE. Values are presented as mean ± SD.


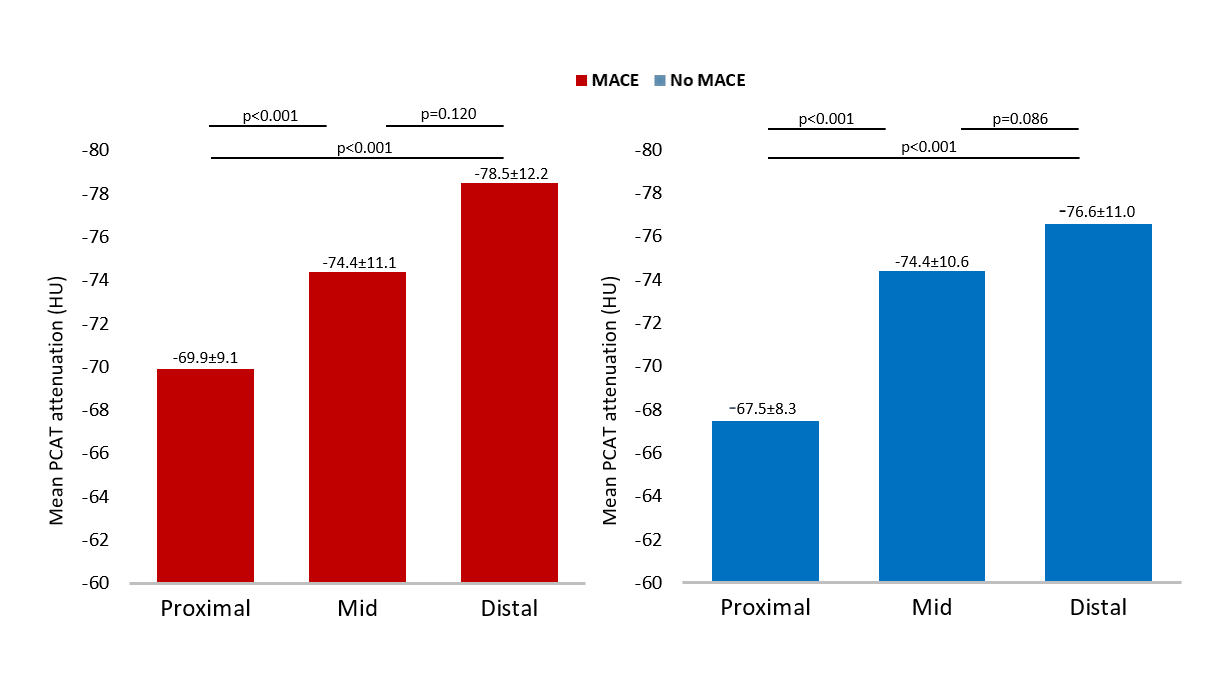


**SUPPLEMENTAL Figure 3.**The association of PCAT attenuation measured in three ways with event-free survival presented by Kaplan-Meier curves and corresponding Log-rank p-values.

**
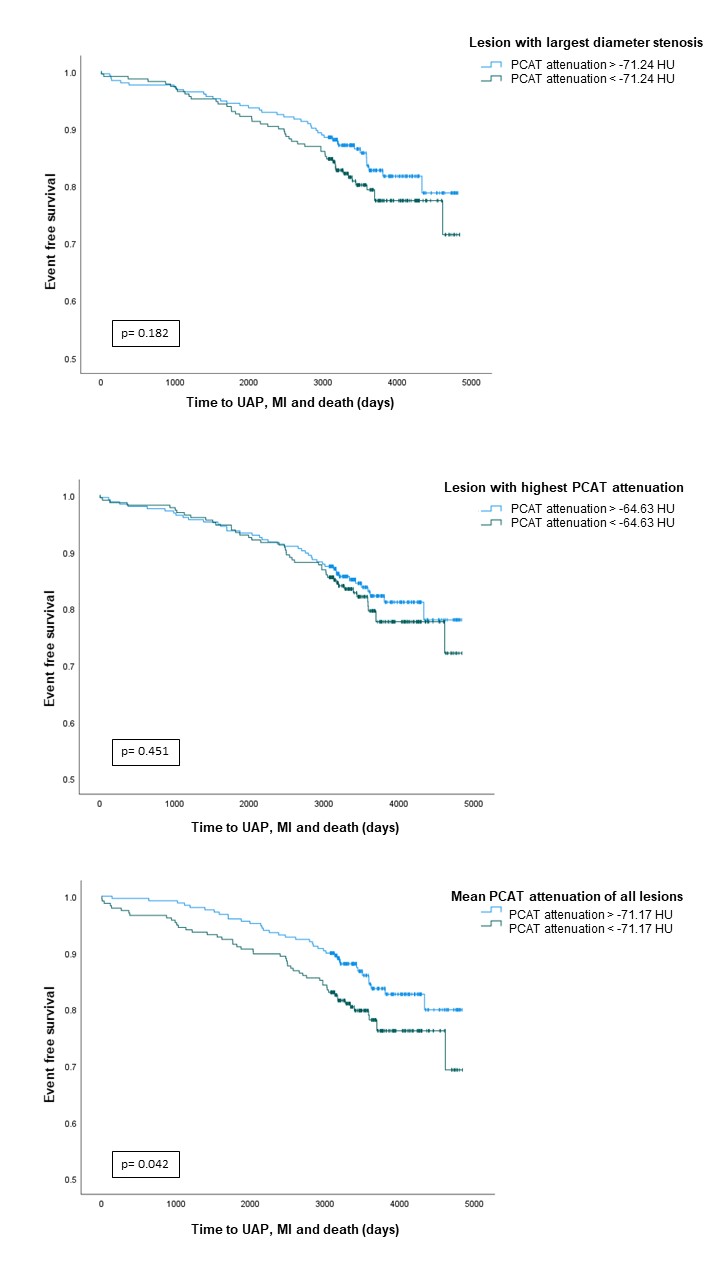
**
